# Oral microbial community composition is associated with pancreatic cancer: A case‐control study in Iran

**DOI:** 10.1002/cam4.2660

**Published:** 2019-11-21

**Authors:** Emily Vogtmann, Yongli Han, J. Gregory Caporaso, Nicholas Bokulich, Ashraf Mohamadkhani, Alireza Moayyedkazemi, Xing Hua, Farin Kamangar, Yunhu Wan, Shalabh Suman, Bin Zhu, Amy Hutchinson, Casey Dagnall, Kristine Jones, Belynda Hicks, Jianxin Shi, Reza Malekzadeh, Christian C. Abnet, Akram Pourshams

**Affiliations:** ^1^ Metabolic Epidemiology Branch Division of Cancer Epidemiology and Genetics National Cancer Institute Bethesda MD USA; ^2^ Biostatistics Branch Division of Cancer Epidemiology and Genetics National Cancer Institute Bethesda MD USA; ^3^ Center for Applied Microbiome Science Pathogen and Microbiome Institute Northern Arizona University Flagstaff AZ USA; ^4^ Digestive Oncology Research Center Digestive Diseases Research Institute Tehran University of Medical Sciences Tehran Iran; ^5^ Department of Internal Medicine Lorestan University of Medical Sciences Khorramabad Iran; ^6^ Liver and Pancreatobiliary Diseases Research Center Digestive Diseases Research Institute Tehran University of Medical Sciences Tehran Iran; ^7^ Department of Biology School of Computer, Mathematical, and Natural Sciences Morgan State University Baltimore MD USA; ^8^ Cancer Genomics Research Laboratory Division of Cancer Epidemiology and Genetics National Cancer Institute Bethesda MD USA; ^9^ Leidos Biomedical Research Laboratory, Inc. Frederick National Laboratory for Cancer Research Frederick MD USA; ^10^ Digestive Disease Research Center Digestive Diseases Research Institute Tehran University of Medical Sciences Tehran Iran

**Keywords:** case‐control study, microbiota, pancreatic cancer

## Abstract

**Background:**

Oral microbiota may be related to pancreatic cancer risk because periodontal disease, a condition linked to multiple specific microbes, has been associated with increased risk of pancreatic cancer. We evaluated the association between oral microbiota and pancreatic cancer in Iran.

**Methods:**

A total of 273 pancreatic adenocarcinoma cases and 285 controls recruited from tertiary hospitals and a specialty clinic in Tehran, Iran provided saliva samples and filled out a questionnaire regarding demographics and lifestyle characteristics. DNA was extracted from saliva and the V4 region of the 16S rRNA gene was PCR amplified and sequenced on the MiSeq. The sequencing data were processed using the DADA2 plugin in QIIME 2 and taxonomy was assigned against the Human Oral Microbiome Database. Logistic regression and MiRKAT models were calculated with adjustment for potential confounders.

**Results:**

No association was observed for alpha diversity with an average of 91.11 (standard deviation [SD] 2.59) sequence variants for cases and 89.42 (SD 2.58) for controls. However, there was evidence for an association between beta diversity and case status. The association between the Bray‐Curtis dissimilarity and pancreatic cancer was particularly strong with a MiRKAT *P*‐value of .000142 and specific principal coordinate vectors had strong associations with cancer risk. Several specific taxa were also associated with case status after adjustment for multiple comparisons.

**Conclusion:**

The overall microbial community appeared to differ between pancreatic cancer cases and controls. Whether these reflect differences evident before development of pancreatic cancer will need to be evaluated in prospective studies.

## Graphical abstract

Specific microbial taxa and periodontal pathogens have been found to be associated with pancreatic cancer in Western populations. In a case‐control study in Iran, we found distinct microbial communities in pancreatic cancer cases and controls in addition to associations with particular microbial taxa.

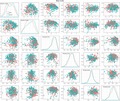

## 1 INTRODUCTION

Microbes living in and on the human body, including bacteria, viruses and archaea, have the potential to impact human health and disease. There is evidence that the microbiota is related to a number of conditions, such as inflammatory bowel disease,^1^ diabetes,^2^ and cancer.^3^ It has been hypothesized that oral microbiota may play a role in the etiology of pancreatic cancer, particularly due to the associations detected between periodontal disease and pancreatic cancer risk.^4^, ^5^, ^6^, ^7^, ^8^, ^9^

Oral health and periodontal disease are associated with oral microbial diversity. Distinct oral microbiome communities by gingivitis status^10^ and dental caries^11^ have been observed. Clustering by periodontal disease status has also been detected^12^ and there are multiple specific microbes strongly implicated in periodontal disease etiology, including *Porphyromonas gingivalis* and *Aggregatibacter actinomycetemcomitans*.^13^ These data suggest that the relationships between oral health and periodontal disease with pancreatic cancer may be related to changes in the oral microbiota. A limited number of studies have assessed the relationship between the oral microbiota or antibodies to oral bacteria and pancreatic cancer.^14^, ^15^, ^16^, ^17^, ^18^ However, these studies were conducted within populations in the United States and Europe and many had very small sample sizes.

Pancreatic cancer ranks as the 12th most common incident cancer globally, but due to its poor prognosis, it is the seventh most common cause of cancer death.^19^ Mortality from pancreatic cancer has also been increasing over the past few years, including in Iran,^20^ unlike trends for many other cancer sites.^21^ Given the poor prognosis after a diagnosis of pancreatic cancer, identifying new risk factors is essential and changes in the oral microbiota offer a promising new avenue in the search for risk factors. Therefore, we evaluated the association between oral microbiota and pancreatic cancer in a case‐control study in Iran.

## 2 METHODS

### 2.1 Study population

The recruitment of pancreatic cancer cases and controls for this study has been previously described in detail.^22^ In brief, participants were recruited from patients referred for endoscopic ultrasonography related to suspicion of a mass or cyst in the pancreas or bile ducts, assessment of submucosal lesions found during esophago‐gastro‐duodenal endoscopy, or to rule out bile duct stones at one of three tertiary hospitals or a specialty clinic in Tehran, Iran from January 2011 to January 2015. After providing informed consent, the participant responded to a questionnaire and provided saliva samples which were immediately stored at −70°C. The questionnaire included information related to demographics, tobacco and opium use, and body mass index. Endoscopic ultrasonography was conducted and for those with mass or cystic lesions, fine needle aspirates were obtained. The pancreatic tissues were interpreted by an expert pathologist and those with pancreatic adenocarcinoma defined by histopathology were considered pancreatic cancer cases. Participants who had a normal pancreas at the endoscopic ultrasonography exam, aged 40 years or older, no history of liver or renal failure or cancer, no consumption of a special diet, and did not develop pancreatic disease or any cancer within one year of the initial visit were considered controls. Final diagnoses for the controls were asymptomatic small (<10 mm) submucosal lesions in the esophagus or stomach, or gallbladder or common bile duct stones without cholangitis. A total of 357 pancreatic cancer cases and 328 controls were identified and saliva specimens were available from 287 cases and 300 controls. Of the 280 cases with staging data, there were 29 stage I, 160 stage II, 37 stage III, and 54 stage IV pancreatic tumors.

### 2.2 DNA extraction, amplification, and sequencing

Saliva samples were shipped on dry ice to the National Cancer Institute for processing. DNA extraction, PCR amplification, and sequencing were completed as described in detail previously.^23^ In brief, DNA extraction batches were created by randomly selecting study participants within sex‐stratified sets of cases and controls to have an adequate distribution of both cases and controls and both sexes within each batch. The laboratory was blinded to the case or control status of each sample. Within each DNA extraction batch, three quality control (QC) samples were also included: oral artificial community or chemostat community^24^; blank; and extraction duplicate of a randomly selected sample. The saliva samples were thawed at 4°C and extracted using the DSP DNA Virus Pathogen kit on a QIAsymphony instrument (Qiagen). The V4 region of the 16S rRNA gene was PCR amplified for 25 cycles and 2 × 250 bp paired end sequencing was performed on the Illumina MiSeq.

### 2.3 Bioinformatic data processing

Sequence data processing was performed with QIIME 2 2017.2.^25^ Sequences were demultiplexed, and quality control and paired‐end read joining were performed with DADA2.^26^ The first ten bases were trimmed from forward and reverse reads; forward reads were truncated at 225 bases and reverse reads were truncated at 200 bases. Taxonomy was assigned to the resulting amplicon sequence variants (ASVs) using q2‐feature‐classifier^27^ and the Human Oral Microbiome Database version 14.51.^28^ ASVs not assigned at least to the phylum level were excluded. Taxonomic relative abundances from the phylum to genus level were generated. A phylogenetic tree was created by aligning ASVs with MAFFT,^29^ filtering highly variable positions using q2‐alignment, and applying FastTree^30^ followed by midpoint rooting using q2‐phylogeny. Diversity metrics, including Bray‐Curtis,^31^ weighted and unweighted UniFrac,^32^ observed sequence variants, Shannon index,^33^ and Faith's Phylogenetic Diversity (PD)^34^ were computed using q2‐diversity at 40 000 sequences per sample. Principal coordinates analysis (PCoA) was applied to the beta diversity distance matrices using q2‐diversity.

For quality control analysis, the taxonomic composition of the oral artificial community samples was compared to the known composition of the mock using q2‐quality‐control. The mean taxon accuracy rate (fraction of observed taxa that were expected^27^) was 0.74 (±0.6 standard deviation [SD]) at the genus level, indicating a low false‐positive error rate. The mean taxon detection rate (fraction of expected taxa that are observed^27^) was 0.77 (±0.3 SD) at the genus level, indicating a low false‐negative detection rate. In addition, both the oral artificial and chemostat communities displayed high levels of consistency across runs with mean Shannon estimates of 3.86 (±0.10 SD) and 4.09 (±0.10 SD), respectively. Similarly, oral artificial community and chemostat community within‐group Bray‐Curtis distances were 0.14 (±0.05 SD) and 0.11 (±0.04 SD), respectively, and significantly different from other sample types (PERMANOVA *P* < .05), indicating a low level of variation in beta diversity across sequencing runs.

### 2.4 Statistical analysis

A total of 273 cases and 285 controls remained after excluding QC samples. Descriptive characteristics of the pancreatic cancer cases and controls were presented. The average alpha diversity estimates were calculated by case status and by demographic and lifestyle factors.

To evaluate associations with pancreatic cancer, we created logistic regression models to calculate odds ratios (OR), 95% confidence intervals (CI), and *P*‐values. Alpha diversity was modeled using quartiles from the distribution within the controls with a test for trend using alpha diversity as a continuous variable. For beta diversity, we modeled the first six PCoA vectors, which accounted for 42%‐65% of the overall variance in the matrices, in independent logistic regression models. The ORs were calculated based on normalized values for the PCoA vectors such that the ORs represent a one standard deviation (SD) increase in the vector. Restricting to taxa with a relative abundance in cases and controls of at least 1%, we created logistic regression models for the relative abundance of taxa. Restricting to taxa with an overall prevalence ranging from 5% to 95%, we created models for the presence of individual taxa. We set Bonferroni‐adjusted *P*‐value significance thresholds based on the number of statistical tests for beta diversity and the taxonomic analyses. For all statistical models, we generated three models: (a) unadjusted; (b) adjusted for age and sex; and (c) adjusted for age, sex, body mass index (BMI; continuous), and any tobacco and/or opium use.

For the association between the overall beta diversity matrices and pancreatic cancer, the MiRKAT test was used.^35^ Additionally, supervised learning was performed with q2‐sample‐classifier^36^ using random forest classification models^37^ grown with 500 trees. Microbial ASV abundances, taxonomic abundances, age, sex, BMI, and tobacco and/or opium use, were used as features for prediction of cancer cases. The model was trained on 80% of the samples and validated on the 20% hold‐out set.

## 3 RESULTS

As seen in Table 1, the pancreatic cancer cases were more likely to be male (60.44%), smoke cigarettes (34.80%), consume alcohol (12.45%), and use opium (16.85%) compared to controls. However, both cases and controls tended to be from an urban residence (65.93% and 66.32%, respectively). Cases and controls had similar average estimates for community richness and evenness (ie, alpha diversity). For example, on average, pancreatic cancer cases had 91.11 observed sequence variants (SD 2.59) compared to 89.42 (SD 2.58) for controls. Multivariable models confirmed the lack of consistent patterns with pancreatic cancer case status for alpha diversity (Table 2). However, alpha diversity was significantly different by age, cigarette smoking, and opium use.

**Table 1:** Description of pancreatic cancer cases and controls from Tehran, Iran, 2011‐2015

	Case		Control		*P*‐value
	N = 273		N = 285		
	Frequency	Percent	Frequency	Percent	
Age group					
<50	26	9.52	20	7.02	.0979
50‐59	61	22.34	78	27.37	
60‐69	87	31.87	94	32.98	
70‐79	77	28.21	59	20.70	
≥80	22	8.06	34	11.93	
Gender					
Male	165	60.44	131	45.96	.0008
Female	108	39.56	154	54.04	
Education					
No formal education	105	38.46	121	42.46	.7216
≤5 y	56	20.51	59	20.70	
6‐8 y	34	12.45	28	9.82	
9‐12 y	40	14.65	44	15.44	
Higher education	38	13.92	33	11.58	
Residence					
Rural	93	34.07	96	33.68	.9954
Urban	180	65.93	189	66.32	
Body mass index					
Underweight (<18)	36	13.19	14	4.91	<.0001
Normal (18‐24.9)	157	57.51	132	46.32	
Overweight (25‐29.9)	64	23.44	97	34.04	
Obese (≥30)	16	5.86	42	14.74	
Any cigarette smoking					
Yes	95	34.80	74	25.96	.0294
No	178	65.20	211	74.04	
Any alcohol consumption					
Yes	34	12.45	8	2.81	<.0001
No	239	87.55	277	97.19	
Any opium use					
Yes	46	16.85	19	6.67	.0003
No	227	83.15	266	93.33	

Note

Any cigarette smoking incorporates reporting ever smoking factory made cigarettes with or without a filter, or smoking hand‐made cigarettes. Any alcohol consumption incorporates reporting ever consuming beer, imported alcoholic beverages, homemade alcoholic beverages, or spirits. Any opium use incorporates reporting ever smoking opium, using heroin, smoking burned opium, using opium juice, or using crystal.

**Table 2:** Association between alpha diversity and principal coordinates of beta diversity matrices with pancreatic cancer from cases and controls in Tehran, Iran, 2011‐2015

Alpha diversity	Model 1		Model 2		Model 3		Model 3 (never cigarettes/opium)	
	OR	95% CI	OR	95% CI	OR	95% CI	OR	95% CI
Observed SVs								
<55	Ref		Ref		Ref		Ref	
55‐86.9	1.56	0.98, 2.50	1.63	1.02, 2.64	1.83	1.12, 3.01	2.02	1.12, 3.67
87‐117.9	1.00	0.61, 1.65	1.06	0.64, 1.76	1.22	0.72, 2.07	0.85	0.43, 1.65
≥118	1.22	0.75, 1.97	1.20	0.73, 1.99	1.49	0.88, 2.55	1.43	0.77, 2.68
*P*‐trend	.6440		.6350		.2066		.3067	
Shannon index								
<3.39	Ref		Ref		Ref		Ref	
3.39‐4.14	1.31	0.83, 2.08	1.33	0.83, 2.12	1.50	0.93, 2.45	1.62	0.89, 2.95
4.15‐4.62	0.97	0.60, 1.56	1.00	0.62, 1.63	1.17	0.70, 1.94	1.19	0.63, 2.26
≥4.63	0.91	0.56, 1.47	0.94	0.57, 1.54	1.12	0.67, 1.88	1.33	0.72, 2.47
*P*‐trend	.7530		.8838		.5747		.3319	
Faith's PD								
<5.07	Ref		Ref		Ref		Ref	
5.07‐6.72	1.10	0.68, 1.77	1.18	0.73, 1.92	1.29	0.78, 2.13	1.67	0.92, 3.05
6.73‐8.41	1.06	0.66, 1.72	1.12	0.69, 1.84	1.27	0.76, 2.13	1.04	0.54, 1.97
≥8.42	1.29	0.81, 2.06	1.33	0.82, 2.16	1.59	0.96, 2.67	1.60	0.86, 3.01
*P*‐trend	.2910		.2892		.0632		.1293	

Beta diversity	Model 1			Model 2			Model 3			Model 3 (never cigarettes/opium)		
	OR	95% CI	*P*‐value	OR	95% CI	*P*‐value	OR	95% CI	*P*‐value	OR	95% CI	*P*‐value
Bray‐Curtis												
PCoA1 (13.8%)	0.73	0.61, 0.86	.0003	0.74	0.62, 0.88	.0006	0.77	0.64, 0.92	.0052	0.70	0.56, 0.87	.0015
PCoA2 (10.5%)	0.79	0.67, 0.93	.0058	0.81	0.68, 0.96	.0146	0.88	0.74, 1.06	.1815	0.91	0.73, 1.12	.3701
PCoA3 (5.7%)	1.09	0.92, 1.29	.3340	1.09	0.92, 1.29	.3240	1.09	0.92, 1.31	.3113	1.37	1.10, 1.73	.0060
PCoA4 (4.7%)	1.26	1.06, 1.49	.0080	1.28	1.08, 1.52	.0054	1.30	1.09, 1.56	.0038	1.41	1.14, 1.77	.0022
PCoA5 (3.9%)	1.02	0.86, 1.20	.8260	1.01	0.85, 1.19	.9412	0.99	0.83, 1.18	.8920	0.92	0.74, 1.13	.4130
PCoA6 (3.4%)	1.21	1.03, 1.44	.0245	1.19	1.00, 1.42	.0480	1.21	1.01, 1.45	.0369	1.08	0.87, 1.34	.4891
Unweighted UniFrac												
PCoA1 (24.4%)	1.05	0.89, 1.24	.5900	1.06	0.89, 1.26	.5358	1.15	0.95, 1.39	.1456	1.16	0.93, 1.46	.1963
PCoA2 (9.4%)	1.32	1.12, 1.57	.0012	1.27	1.07, 1.51	.0067	1.23	1.03, 1.47	.0259	1.24	1.00, 1.53	.0510
PCoA3 (4.6%)	0.71	0.60, 0.85	.0001	0.71	0.60, 0.85	.0001	0.74	0.61, 0.88	.0009	0.61	0.49, 0.77	<.0001
PCoA4 (3.3%)	1.03	0.87, 1.22	.7190	1.05	0.88, 1.24	.5873	1.09	0.91, 1.30	.3550	1.06	0.86, 1.31	.5935
PCoA5 (3.0%)	0.99	0.84, 1.17	.9180	1.02	0.86, 1.21	.8359	1.07	0.90, 1.28	.4530	1.15	0.93, 1.43	.1916
PCoA6 (2.1%)	1.08	0.92, 1.28	.3400	1.08	0.91, 1.28	.3677	1.13	0.95, 1.35	.1708	1.00	0.81, 1.24	.9955
Weighted UniFrac												
PCoA1 (31.2%)	0.84	0.71, 0.99	.0398	0.86	0.72, 1.02	.0841	0.93	0.78, 1.12	.4585	1.01	0.81, 1.25	.9560
PCoA2 (14.4%)	0.75	0.63, 0.89	.0014	0.75	0.63, 0.90	.0018	0.78	0.65, 0.94	.0087	0.64	0.50, 0.80	.0002
PCoA3 (6.8%)	0.86	0.73, 1.02	.0824	0.86	0.73, 1.02	.0939	0.84	0.70, 1.00	.0509	0.86	0.70, 1.07	.1763
PCoA4 (5.2%)	1.11	0.94, 1.32	.2050	1.12	0.95, 1.33	.1846	1.16	0.97, 1.39	.0961	1.26	1.02, 1.58	.0391
PCoA5 (3.9%)	1.04	0.88, 1.24	.6040	1.05	0.89, 1.24	.5851	1.02	0.85, 1.22	.8277	1.02	0.82, 1.26	.8578
PCoA6 (3.4%)	1.12	0.95, 1.33	.1830	1.13	0.96, 1.34	.1496	1.14	0.96, 1.36	.1397	1.24	1.00, 1.55	.0504

Note

Model 1: Unadjusted.

Model 2: Adjusted for age (continuous) and sex.

Model 3: Adjusted for age, sex, BMI (continuous), and any tobacco or opium use.

Model 3 (never cigarettes/opium): Adjusted for age, sex, and BMI (continuous) within individuals who reported never using cigarettes or opium.

ORs for beta diversity PCoA vectors are calculated based on a one standard deviation increase in the PCoA vector.

Bonferroni adjusted *P*‐value significance threshold for beta diversity comparisons was *P* < .05/6 = 0.0083.

When considering the overall microbial community composition, as captured by the full beta diversity matrices, a significant difference was detected between pancreatic cancer cases and controls. For example, in unadjusted MiRKAT models, the *P*‐value for the association with case status was 5.15 × 10^−7^ for Bray‐Curtis. After adjustment for age, sex, BMI (continuous), and use of tobacco or opium, the associations with beta diversity were attenuated, but remained statistically significant (Table 3). Random Forest classification could weakly predict pancreatic cancer based on the oral microbiota and other covariates, yielding a 32.1% error rate for prediction of cases compared to controls, a reduction from the baseline error rate of 49.1%. The top 25 most predictive features included several ASVs and taxa including *Streptococcus parasanguinis*, *Granulicatella adiacens*, and *Rothia aeria*.

**Table 3:** MiRKAT test for association between beta diversity matrices and pancreatic cancer case status

*P* values	Model 1	Model 2	Model 3
Bray‐Curtis	5.15E‐07	2.94E‐06	1.42E‐04
Weighted UniFrac	1.40E‐03	3.50E‐03	2.67E‐02
Unweighted UniFrac	6.95E‐03	1.11E‐02	1.05E‐02

Note

Model 1: Unadjusted.

Model 2: Adjusted for age (continuous) and sex.

Model 3: Adjusted for age, sex, BMI (continuous), and any tobacco or opium use.

To further understand the overall beta diversity association with pancreatic cancer, we investigated the first six PCoA vectors from the three beta diversity matrices. No visual clustering was observed by case status, but strong associations were observed for specific PCoA vectors in logistic regression models. For the Bray‐Curtis matrix, two PCoA vectors were significantly associated with pancreatic cancer after adjustment for potential confounders. For unweighted UniFrac and weighted UniFrac, one PCoA vector each was associated with pancreatic cancer in the fully adjusted models, although the *P*‐value for PCoA2 from weighted UniFrac was marginally higher than the Bonferroni‐adjusted significance threshold. In general, associations were similar when restricted to individuals who reported never smoking cigarettes or using opium (Table 2).

From a single beta diversity matrix, the PCoA vectors are orthogonal and therefore uncorrelated with other PCoA vectors, but this is not the case for PCoA vectors from different beta diversity matrices. To evaluate whether the PCoA associations from each beta diversity matrix were measuring similar microbial community characteristics, we calculated Pearson correlations between the vectors. Bray‐Curtis PCoA1 was positively correlated with both unweighted UniFrac PCoA3 (R = 0.39) and weighted UniFrac PCoA2 (R = 0.53), while Bray‐Curtis PCoA4 was negatively correlated with both unweighted UniFrac PCoA3 (R = −0.23) and weighted UniFrac PCoA2 (R = −0.43). Unweighted UniFrac PCoA3 was also positively correlated with weighted UniFrac PCoA2 (R = 0.37). The significant PCoA vectors were also associated with some demographic and lifestyle factors. For example, Bray‐Curtis PCoA1 was associated with sex, cigarette smoking, and opium use, but as noted above, these vectors remained significantly associated with pancreatic cancer case status in fully adjusted models.

When considering individual taxa, for relative abundance, only one genus had a marginally significant association with the odds of pancreatic cancer, *Haemophilus*. The OR for an increase of 1% in the relative abundance of *Haemophilus* was 0.95 (95% CI: 0.92, 0.98), although the *P*‐value was slightly greater than the Bonferroni‐adjusted significance threshold of 0.0038 (*P* = .0043). However, we saw consistently strong inverse associations at higher taxonomic levels for *Haemophilus* (ie, *Proteobacteria*, *Gammaproteobacteria*, *Pasteurellales*, and *Pasteurellaceae*, at the phylum, class, order, and family levels, respectively) demonstrating the overall lower odds of pancreatic cancer at all taxonomic levels. For the presence/absence of taxa, for individuals with presence of the order *Enterobacteriales*, there was 2.80 times greater odds of pancreatic cancer (95% CI: 1.69, 4.78). This strong association was seen at the family level (*Enterobacteriaceae*) and at the genus level (unknown genus). Similarly, the presence of the genus *Lachnospiraceae G7* was associated with 2.40 times greater odds of pancreatic cancer (95% CI: 1.52, 3.84). Although not statistically significant using a Bonferroni‐adjusted *P*‐value significance threshold of .0011, the presence of the family *Bacteroidaceae* (OR 1.90; 95% CI: 1.29, 2.84) or *Staphylococcaceae* (OR 1.81; 95% CI: 1.25, 2.62) were both associated with increased odds of pancreatic cancer. When we restricted to individuals who reported never smoking cigarettes or using opium, the association estimates for the statistically significant taxa from both relative abundance and the presence/absence were generally similar (results not shown).

## 4 DISCUSSION

In this study of 273 pancreatic cancer cases and 285 controls from Iran, we found evidence of differing microbial communities by case status using multiple methods to test beta diversity. No associations with pancreatic cancer were detected for alpha diversity. There were also indications for associations between specific taxa and pancreatic cancer. Increasing relative levels of *Haemophilus* were associated with decreased odds of pancreatic cancer, while the presence of *Enterobacteriaceae*, *Lachnospiraceae G7*, *Bacteroidaceae*, or *Staphylococcaceae* were associated with increased odds of pancreatic cancer.

In previous studies of the oral microbiota and pancreatic cancer, no associations between alpha diversity and pancreatic cancer have been detected^15^, ^18^ which was consistent with our findings. This is notable given the possibility of reverse causation in a cross‐sectional study of pancreatic cancer where subjects may be feeling unwell at the time of enrolment. Lower alpha diversity in fecal samples has been found to be related to inflammatory bowel disease,^1^ colorectal cancer,^38^ and Westernization,^39^ which suggests that increased fecal microbial diversity is a marker of good overall health. Oral microbial diversity may be less indicative of general overall health and for pancreatic cancer, not a marker of disease.

We found associations between pancreatic cancer and the microbial community composition (ie, beta diversity), but previous studies have not observed this association.^15^, ^16^, ^18^ Analyses with beta diversity are complicated due to the pairwise nature of beta diversity matrices and PCoA vectors are dependent on the distance matrix from the study population and not directly comparable to other studies. Methods are being developed to facilitate between study comparisons by using a standard reference set from which to calculate dissimilarities,^40^ but no ideal standard reference set currently exists. Our beta diversity associations may differ from previous studies because they focused on Western populations. A recent study observed that within one province in China, the factor that explained the largest percent of variability in beta diversity was the district in which the individual lived. In addition, microbial models to predict specific diseases were location specific.^41^ The beta diversity associations we observed may be specific to the Iranian population or associations may be detected using a standard reference set.

Previous studies have also investigated associations between specific bacteria or antibodies to bacteria with pancreatic cancer. We found that an increased relative abundance of *Haemophilus* and its higher taxonomic levels (ie, *Proteobacteria*, *Gammaproteobacteria*, *Pasteurellales*, and *Pasteurellaceae*) were associated with decreased odds of pancreatic cancer. Two previous studies also observed lower relative abundances of *Proteobacteria* in pancreatic cancer cases compared to controls^15^, ^18^ with one finding an association with *Haemophilus*.^18^ The presence of *Enterobacteriaceae*, *Lachnospiraceae G7*, *Bacteroidaceae*, or *Staphylococcaceae* were associated with increased odds of pancreatic cancer, although *Bacteroidaceae* and *Staphylococcaceae* had slightly greater *P*‐values than the Bonferroni‐adjusted threshold. One study found that individuals who had pancreatic tissue sampled after foregut surgery had a higher prevalence of *Kluyvera* (genus within the *Enterobacteriaceae* family) compared to pancreatic tissue from deceased controls; however, when comparing pancreatic cancer tissue to deceased control pancreatic tissue, *Salmonella*, *Enterobacter*, and *Raoultella* (genera within the *Enterobacteriaceae* family) had a lower prevalence. Similar to our results, this study also found a higher prevalence of *Bacteroides* (genus within the *Bacteroidaceae* family) in pancreatic cancer tissue compared to deceased control pancreatic tissue.^42^

A number of additional taxa have been detected to be associated with pancreatic cancer in previous studies, often with a special focus on periodontal pathogens.^4^, ^5^, ^6^ *P gingivalis*, antibody detected and from oral wash specimens, was associated with increased risk of pancreatic cancer in two prospective studies.^16^, ^17^ However, a small case‐control study found lower levels of *Porphyromonas* in oral samples from pancreatic cancer cases compared to controls.^15^ In our study, *Porphyromonas* was not associated with pancreatic cancer and was detected in 76.92% of cases and 76.49% of controls. The presence of another periodontal pathogen, *Aggregatibacter actinomycetemcomitans*, was associated with an increased risk of pancreatic cancer, but not other periodontal pathogens (*Tannerella forsythia* and *Prevotella intermedia*).^16^ The periodontal pathogen association differences could again be related to the distinct population under study since there are differences in oral health in Iran compared to Western populations. One estimate of tooth loss from the Golestan Cohort study, a cohort of adults in northeastern Iran, found extensive tooth loss with men and women having lost an average of 15.9 and 18.7 teeth, respectively.^43^ In comparison, estimates from NHANES in the United States indicated that adults aged 50 to 64 had on average lost only about 5.7 teeth.^44^ It is also possible that some associations may differ due to unique oral sampling methods since previous studies have observed clustering by oral site or collection method.^45^, ^46^, ^47^

This study has some limitations. First, saliva samples were collected from pancreatic cancer cases at the time of diagnosis, so we cannot distinguish whether any microbial associations were related to pancreatic cancer etiology or presence of disease. Second, controls were identified within patients who were also referred for endoscopic ultrasonography, thereby not representing healthy individuals who may have experienced microbial changes from underlying conditions. Third, although we had a fairly large sample size compared to the previous studies, this study was still underpowered for the taxa‐specific analyses. Finally, information regarding oral health or tooth loss in this population was not obtained so we are unable to address potential confounding by these factors. However, to the best of our knowledge, this is the first study of the oral microbiota and pancreatic cancer conducted outside of the United States or Europe.

In conclusion, the oral microbial communities detected in pancreatic cancer cases differed from controls. The presence or relative abundance of some specific microbial taxa were also associated with pancreatic cancer, including *Haemophilus*, *Enterobacteriaceae*, *Lachnospiraceae G7*, *Bacteroidaceae*, and *Staphylococcaceae*. The microbial community and taxa‐level differences could be related to the presence of pancreatic cancer or the risk of developing pancreatic cancer. Therefore, we need large, prospective studies of diverse populations to evaluate these associations, both for determining the microbiota related to pancreatic cancer etiology, but also for identifying microbiota that may help with early detection.

## CONFLICT OF INTEREST

We have no competing interests to report.

## AUTHOR CONTRIBUTIONS

EV, FK, RM, CCA, and AP made substantial contributions to the conception or design of the work. EV, YH, JGC, NB, AM, AM, XH, YW, SS, BZ, AH, CD, KJ, BH, SH, RM, CCA, and AP involved in acquisition, analysis, or interpretation of data for the work. EV, YH, JGC, and NB involved in drafting the work. EV, YH, JGC, NB, AM, AM, XH, FK, YW, SS, BZ, AH, CD, KJ, BH, JS, RM, CCA, and AP involved in critical revision of the work for important intellectual content, and all authors gave final approval of the version to be published and agreed to be accountable for all aspects of the work.

## Data Availability

The sequencing data is available on the Sequence Read Archive (NCBI SRA) under BioProject ID PRJNA549488).

## References

[1] Kostic AD , Xavier RJ , Gevers D . The microbiome in inflammatory bowel disease: current status and the future ahead. Gastroenterology. 2014;146:1489‐1499. 24560869 10.1053/j.gastro.2014.02.009 PMC4034132

[2] Forslund K , Hildebrand F , Nielsen T , et al. Disentangling type 2 diabetes and metformin treatment signatures in the human gut microbiota. Nature. 2015;528:262‐266. 26633628 10.1038/nature15766 PMC4681099

[3] Vogtmann E , Goedert JJ . Epidemiologic studies of the human microbiome and cancer. Br J Cancer. 2016;114:237‐242. 26730578 10.1038/bjc.2015.465 PMC4742587

[4] Michaud DS , Liu Y , Meyer M , Giovannucci E , Joshipura K . Periodontal disease, tooth loss, and cancer risk in male health professionals: a prospective cohort study. Lancet Oncol. 2008;9:550‐558. 18462995 10.1016/S1470-2045(08)70106-2 PMC2601530

[5] Ahn J , Segers S , Hayes RB . Periodontal disease, *Porphyromonas gingivalis* serum antibody levels and orodigestive cancer mortality. Carcinogenesis. 2012;33:1055‐1058. 22367402 10.1093/carcin/bgs112 PMC3334514

[6] Stolzenberg‐Solomon RZ , Dodd KW , Blaser MJ , Virtamo J , Taylor PR , Albanes D . Tooth loss, pancreatic cancer, and *Helicobacter pylori* . Am J Clin Nutr. 2003;78:176‐181. 12816788 10.1093/ajcn/78.1.176

[7] Hiraki A , Matsuo K , Suzuki T , Kawase T , Tajima K . Teeth loss and risk of cancer at 14 common sites in Japanese. Cancer Epidemiol Biomarkers Prev. 2008;17:1222‐1227. 18483345 10.1158/1055-9965.EPI-07-2761

[8] Hujoel PP , Drangsholt M , Spiekerman C , Weiss NS . An exploration of the periodontitis‐cancer association. Ann Epidemiol. 2003;13:312‐316. 12821269 10.1016/s1047-2797(02)00425-8

[9] Michaud DS , Kelsey KT , Papathanasiou E , Genco CA , Giovannucci E . Periodontal disease and risk of all cancers among male never smokers: an updated analysis of the Health Professionals Follow‐up Study. Ann Oncol. 2016;27:941‐947. 26811350 10.1093/annonc/mdw028 PMC4843185

[10] Huang S , Yang F , Zeng X , et al. Preliminary characterization of the oral microbiota of Chinese adults with and without gingivitis. BMC Oral Health. 2011;11:33. 22152152 10.1186/1472-6831-11-33 PMC3254127

[11] Yang F , Zeng X , Ning K , et al. Saliva microbiomes distinguish caries‐active from healthy human populations. ISME J. 2012;6:1‐10. 21716312 10.1038/ismej.2011.71 PMC3246229

[12] Liu B , Faller LL , Klitgord N , et al. Deep sequencing of the oral microbiome reveals signatures of periodontal disease. PLoS ONE. 2012;7:e37919. 22675498 10.1371/journal.pone.0037919 PMC3366996

[13] Consensus report. Periodontal diseases: pathogenesis and microbial factors. Ann Periodontol. 1996;1:926‐932. 9118284 10.1902/annals.1996.1.1.926

[14] Farrell JJ , Zhang L , Zhou H , et al. Variations of oral microbiota are associated with pancreatic diseases including pancreatic cancer. Gut. 2012;61:582‐588. 21994333 10.1136/gutjnl-2011-300784 PMC3705763

[15] Torres PJ , Fletcher EM , Gibbons SM , Bouvet M , Doran KS , Kelley ST . Characterization of the salivary microbiome in patients with pancreatic cancer. PeerJ. 2015;3:e1373. 26587342 10.7717/peerj.1373 PMC4647550

[16] Fan X , Alekseyenko AV , Wu J , et al. Human oral microbiome and prospective risk for pancreatic cancer: a population‐based nested case‐control study. Gut. 2018;67:120‐127. 27742762 10.1136/gutjnl-2016-312580 PMC5607064

[17] Michaud DS , Izard J , Wilhelm‐Benartzi CS , et al. Plasma antibodies to oral bacteria and risk of pancreatic cancer in a large European prospective cohort study. Gut. 2013;62:1764‐1770. 22990306 10.1136/gutjnl-2012-303006 PMC3815505

[18] Olson SH , Satagopan J , Xu Y , et al. The oral microbiota in patients with pancreatic cancer, patients with IPMNs, and controls: a pilot study. Cancer Causes Control. 2017;28:959‐969. 28762074 10.1007/s10552-017-0933-8 PMC5709151

[19] Ferlay J , Soerjomataram I , Dikshit R , et al. Cancer incidence and mortality worldwide: sources, methods and major patterns in GLOBOCAN 2012. Int J Cancer. 2015;136:E359‐E386. 25220842 10.1002/ijc.29210

[20] Salimzadeh H , Delavari F , Sauvaget C , et al. Annual trends of gastrointestinal cancers mortality in Iran during 1990–2015. NASBOD Study. Arch Iran Med. 2018;21:46‐55. 29664654

[21] Lucas AL , Malvezzi M , Carioli G , et al. Global trends in pancreatic cancer mortality from 1980 through 2013 and predictions for 2017. Clin Gastroenterol Hepatol. 2016;14:1452‐1462.e1454. 27266982 10.1016/j.cgh.2016.05.034 PMC5028258

[22] Shakeri R , Kamangar F , Mohamadnejad M , et al. Opium use, cigarette smoking, and alcohol consumption in relation to pancreatic cancer. Medicine (Baltimore). 2016;95:e3922. 27428185 10.1097/MD.0000000000003922 PMC4956779

[23] Vogtmann E , Hua X , Zhou L , et al. Temporal variability of oral microbiota over 10 months and the implications for future epidemiologic studies. Cancer Epidemiol Biomark Prev. 2018;27:594‐600. 10.1158/1055-9965.EPI-17-1004 PMC5932224 29475969

[24] Sinha R , Abu‐Ali G , Vogtmann E , et al. Assessment of variation in microbial community amplicon sequencing by the Microbiome Quality Control (MBQC) project consortium. Nat Biotechnol. 2017;35:1077‐1086. 28967885 10.1038/nbt.3981 PMC5839636

[25] Bolyen E , Rideout JR , Dillon MR , et al. Reproducible, interactive, scalable and extensible microbiome data science using QIIME 2. Nat Biotechnol. 2019;37:852‐857. 31341288 10.1038/s41587-019-0209-9 PMC7015180

[26] Callahan BJ , McMurdie PJ , Rosen MJ , Han AW , Johnson AJ , Holmes SP . DADA2: High‐resolution sample inference from illumina amplicon data. Nat Methods. 2016;13:581‐583. 27214047 10.1038/nmeth.3869 PMC4927377

[27] Bokulich NA , Kaehler BD , Rideout JR , et al. Optimizing taxonomic classification of marker‐gene amplicon sequences with QIIME 2's q2‐feature‐classifier plugin. Microbiome. 2018;6:90. 29773078 10.1186/s40168-018-0470-z PMC5956843

[28] Chen T , Yu WH , Izard J , Baranova OV , Lakshmanan A , Dewhirst FE . The human oral microbiome database: a web accessible resource for investigating oral microbe taxonomic and genomic information. Database (Oxford). 2010;2010:baq013. 20624719 10.1093/database/baq013 PMC2911848

[29] Katoh K , Standley DM . MAFFT multiple sequence alignment software version 7: improvements in performance and usability. Mol Biol Evol. 2013;30:772‐780. 23329690 10.1093/molbev/mst010 PMC3603318

[30] Price MN , Dehal PS , Arkin AP . FastTree 2–approximately maximum‐likelihood trees for large alignments. PLoS ONE. 2010;5:e9490. 20224823 10.1371/journal.pone.0009490 PMC2835736

[31] Sprense T . A method of establishing groups of equal amplitude in plant sociology based on similarity of species content and its application to analysis of the vegetation on Danish commons. Biol Skr. 1948;5:1‐34.

[32] Lozupone C , Knight R . UniFrac: a new phylogenetic method for comparing microbial communities. Appl Environ Microbiol. 2005;71:8228‐8235. 16332807 10.1128/AEM.71.12.8228-8235.2005 PMC1317376

[33] Shannon CE . A mathematical theory of communication. Bell Syst Tech J. 1948;27(379–423):623‐656.

[34] Faith DP . Conservation evaluation and phylogenetic diversity. Biol Cons. 1992;61:1‐10.

[35] Zhao N , Chen J , Carroll IM , et al. Testing in microbiome‐profiling studies with MiRKAT, the microbiome regression‐based kernel association test. Am J Hum Genet. 2015;96:797‐807. 25957468 10.1016/j.ajhg.2015.04.003 PMC4570290

[36] Bokulich N , Dillon M , Bolyen E , Kaehler BD , Huttley GA , Caporaso JG . q2‐sample‐classifier: machine‐learning tools for microbiome classification and regression. BioRxiv. 2018:306167. 10.21105/joss.00934 PMC6759219 31552137

[37] Breiman L . Random forests. Mach Learn. 2001;45:5‐32.

[38] Ahn J , Sinha R , Pei Z , et al. Human gut microbiome and risk for colorectal cancer. J Natl Cancer Inst. 2013;105:1907‐1911. 24316595 10.1093/jnci/djt300 PMC3866154

[39] Yatsunenko T , Rey FE , Manary MJ , et al. Human gut microbiome viewed across age and geography. Nature. 2012;486:222‐227. 22699611 10.1038/nature11053 PMC3376388

[40] Maziarz M , Pfeiffer RM , Wan Y , Gail MH . Using standard microbiome reference groups to simplify beta‐diversity analyses and facilitate independent validation. Bioinformatics. 2018;34:3249‐3257. 29668831 10.1093/bioinformatics/bty297 PMC6157088

[41] He Y , Wu W , Zheng HM , et al. Regional variation limits applications of healthy gut microbiome reference ranges and disease models. Nat Med. 2018;24:1532‐1535. 30150716 10.1038/s41591-018-0164-x

[42] Del Castillo E , Meier R , Chung M , et al. The microbiomes of pancreatic and duodenum tissue overlap and are highly subject specific but differ between pancreatic cancer and noncancer subjects. Cancer Epidemiol Biomarkers Prev. 2019;28:370‐383. 30373903 10.1158/1055-9965.EPI-18-0542 PMC6363867

[43] Vogtmann E , Etemadi A , Kamangar F , et al. Oral health and mortality in the Golestan Cohort Study. Int J Epidemiol. 2017;46:2028‐2035. 28449082 10.1093/ije/dyx056 PMC5837566

[44] National Institute of Dental and Craniofacial Research . Tooth loss in adults (age 20 to 64). https://www.nidcr.nih.gov/research/data-statistics/tooth-loss/adults. Accessed March 28, 2019.

[45] Segata N , Haake SK , Mannon P , et al. Composition of the adult digestive tract bacterial microbiome based on seven mouth surfaces, tonsils, throat and stool samples. Genome Biol. 2012;13:R42. 22698087 10.1186/gb-2012-13-6-r42 PMC3446314

[46] Yu G , Phillips S , Gail MH , et al. Evaluation of buccal cell samples for studies of oral microbiota. Cancer Epidemiol Biomarkers Prev. 2017;26:249‐253. 27770008 10.1158/1055-9965.EPI-16-0538

[47] Vogtmann E , Chen J , Kibriya MG , et al. Comparison of oral collection methods for studies of microbiota. Cancer Epidemiol Biomarkers Prev. 2019;28:137‐143. 30262598 10.1158/1055-9965.EPI-18-0312 PMC6324947

